# The impact of a short‐term high‐fat diet on mitochondrial respiration, reactive oxygen species production, and dynamics in oxidative and glycolytic skeletal muscles of young rats

**DOI:** 10.14814/phy2.13548

**Published:** 2018-02-26

**Authors:** Jean‐Philippe Leduc‐Gaudet, Olivier Reynaud, François Chabot, Jocelyne Mercier, David E. Andrich, David H. St‐Pierre, Gilles Gouspillou

**Affiliations:** ^1^ Département des Sciences de l'activité physique Faculté des Sciences UQAM Montréal Canada; ^2^ Groupe de recherche en Activité Physique Adaptée Montréal Canada; ^3^ Meakins‐Christie Laboratories Department of Medicine and Division of Experimental Medicine McGill University Québec Canada; ^4^ Centre de Recherche du CHU Sainte‐Justine Montréal Canada; ^5^ Centre de Recherche de l'Institut Universitaire de Gériatrie de Montréal Montréal Canada

**Keywords:** High‐fat feeding, lipid metabolism, mitochondria, mitochondrial fission

## Abstract

Multiple aspects of mitochondrial function and dynamics remain poorly studied in the skeletal muscle of pediatric models in response to a short‐term high‐fat diet (HFD). This study investigated the impact of a short‐term HFD on mitochondrial function and dynamics in the oxidative soleus (SOL) and glycolytic extensor digitorum longus (EDL) muscles in young rats. Young male Wistar rats were submitted to either HFD or normal chow (NCD) diets for 14 days. Permeabilized myofibers from SOL and EDL were prepared to assess mitochondrial respiration and reactive oxygen species (ROS) production. The expression and content of protein involved in mitochondrial metabolism and dynamics (fusion/fission) were also quantified. While no effects of HFD was observed on mitochondrial respiration when classical complex I and II substrates were used, both SOL and EDL of rats submitted to a HFD displayed higher basal and ADP‐stimulated respiration rates when Malate + Palmitoyl‐L‐carnitine were used as substrates. HFD did not alter ROS production and markers of mitochondrial content. The expression of CPT1b was significantly increased in SOL and EDL of HFD rats. Although the expression of UCP3 was increased in SOL and EDL muscles from HFD rats, mitochondrial coupling efficiency was not altered. In SOL of HFD rats, the transcript levels of Mfn2 and Fis1 were significantly upregulated. The expression and content of proteins regulating mitochondrial dynamics was not modulated by HFD in the EDL. Finally, DRP1 protein content was increased by over fourfold in the SOL of HFD rats. Taken altogether, our findings show that exposing young animals to short‐term HFD results in an increased capacity of skeletal muscle mitochondria to oxidize fatty acids, without altering ROS production, coupling efficiency, and mitochondrial content. Our results also highlight that the impact of HFD on mitochondrial dynamics appears to be muscle specific.

## Introduction

Obesity is one of the major risk factors for the development of metabolic disorders such as insulin resistance, type 2 diabetes Mellitus (T2DM), nonalcoholic liver disease, and atherosclerosis (Dedoussis et al. 2007). Because obesity is strongly associated with excessive energy consumption, feeding high‐fat diets (HFD) to rodents were extensively studied to investigate the etiology of metabolic disturbances (Hariri and Thibault 2010). Among the underlying mechanisms, fat accumulation in muscle cells were shown to interfere with insulin signaling pathways, and therefore contribute to insulin resistance (Brons and Vaag 2009). Although controversies still remain, the accumulation of mitochondrial dysfunctions, including reactive oxygen species (ROS) over production, impaired energetics, and a reduced ability to oxidize fat, has been proposed to play a major role in this process (Anderson et al. 2009; Morrow et al. 2017). Because until recently, T2DM was mainly prevalent in individuals over 50, most of the studies investigating the effects of HFD on mitochondrial function were performed in adult animals. However, with the important alterations in lifestyles and nutritional habits observed, the prevalence of pediatric obesity and metabolic disorders increased drastically over the last few decades. Furthermore, the incidence of obesity is now estimated at 17% (Pulgaron and Delamater 2014), whereas cases of T2DM increased by 14% between 2000 and 2008 in US children and adolescents (May et al. 2012). Because of this alarming epidemiological trend, there is a critical need to determine the effects of HFD on mitochondrial function in the skeletal muscle using relevant pediatric animal models. This is further supported by the fact that mitochondrial dysfunction were reported in the skeletal muscle of insulin‐resistant obese adolescents (Slattery et al. 2014).

To date, only few studies investigated the impact of short‐term HFD on indices of mitochondrial function in skeletal muscles of young, developing animals. (Thomas et al. (2014) reported that while the activity of the mitochondrial enzyme succinate dehydrogenase stain was increased, lipid oxidation was not altered in the soleus of 3‐week‐old mice submitted to HFD for 3 weeks. In contrast, Hancock et al. showed increases in markers of mitochondrial content and palmitate oxidation in skeletal muscle in response to 4–5 weeks of HFD in young rats (50 g) (Hancock et al. 2008). Furthermore, Li et al. (2016) observed a significant increase in citrate synthase and beta‐hydroxyacyl CoA dehydrogenase activities in the *plantaris* muscle of 4‐week‐old rats fed with HFD for 4 weeks. Taken together, current state of knowledge suggests that short‐term HFD increases mitochondrial enzyme activities and mitochondrial content in young animal, although its functional impact on lipid oxidation remains controversial (Thomas et al. 2014; Hancock et al. 2008; Li et al. 2016). It is also important to highlight that the impact of HFD on mitochondrial ROS production was not previously investigated in young animals. Because mitochondrial ROS overproduction is often proposed as a mechanism promoting insulin resistance in skeletal muscles of adults (Anderson et al. 2009; Di Meo et al. 2017), it is of utmost importance to clarify this issue.

It was recently proposed that impairments in mitochondrial dynamics could influence the development of insulin resistance in adult animals submitted to HFD (Bach et al. 2005; Chan 2012; Cheng and Almeida 2014; Sebastian et al. 2012; Putti et al. 2015; Jheng et al. 2012). The term mitochondrial dynamics regroups processes through which mitochondria undergo fusion and fission. In the mitochondria, fusion involves Mfn2 and optic atrophy 1 (OPA) (1) while fission is regulated by dynamin‐related protein 1 (Drp1) and mitochondrial fission 1 protein (Fis1) (Chan 2006). Interestingly, Sebastian et al. (2012) reported that the expression of the profusion protein Mfn2 is downregulated in skeletal muscles of adult rats fed with HFD. Also, mice with an inactivated form of the Mfn2 gene display impaired insulin sensitivity. Furthermore, Jheng et al. (2012) reported that adult mice submitted to HFD display smaller and shorter mitochondria in the skeletal muscle. This indicates that HFD could therefore trigger mitochondrial fragmentation. However, the impact of HFD on mitochondrial dynamics in young, developing muscle, remains to be established.

The aim of this study was therefore to investigate the early effects of HFD on mitochondrial bioenergetics, ROS production and dynamics in oxidative soleus (SOL) and glycolytic *extensor digitorum longus* (EDL) muscles of young rats.

## Methods

### Animals

All experiments were approved by the Animal Care Committee (CIPA) of *Université du Québec à Montréal* (UQAM) (Permit Number: 0515‐R3‐759‐0516). Forty young male Wistar rats (~150 g; 37–40 days; purchased from Charles River, St‐Constant, QC), were included in the experimental protocol after 3 days of acclimatization at UQAM's animal facility. Rats were randomized to two individual weight‐balanced groups: normal chow diet (NCD, *n *= 20) or HFD (*n *= 20). All rats were housed individually (at 24 ± 1°C, 50–60% relative humidity), on a 12 h light/dark cycle and fed ad libitum. The first group was submitted to NCD (Charles River Rodent Diet # 5075, Cargill Animal Nutrition, Minnetonka, MN) with a physiological fuel value of 2.89 kcal/g calculated using modified Atwater factors (21.4% of energy from protein, 65.6% of energy from carbohydrates, 13% of energy from fat) (see Table S1 for more details). The second group submitted to HFD prepared from purified food‐grade elements according to a commercial diet (D12492 diet, Research Diets Inc.). This HFD contained a physiological fuel value 4.80 kcal/g calculated from modified Atwater factors (19% of energy from protein, 19.2% of energy from carbohydrates, 61.8% of energy from fat). The HFD diet protein sources were casein and L‐cystine (98.5% and 1.5%, respectively), lipid sources were lard and soybean oil (90.7% and 9.3%, respectively), whereas carbohydrate sources were maltodextrin and sucrose (64.5% and 35.5%, respectively). The diet also contained cellulose (64.6 g/kg), calcium carbonate (7.1 g/kg), dicalcium phosphate (16.8 g/kg), potassium citrate (21.3 g/kg), and choline bitartrate (2.6 g/kg) as well as mineral (12.9 g/kg) and vitamin (12.9 g/kg) mixes (see Table S1 for more details).

### In vivo assessment of rat metabolic profile

Rats were single‐housed in metabolic measurement cages (CLAMS from Oxymax instrument, Columbus Instruments, OH) for the duration of the experiment. Data collection for oxygen consumption (VO_2_), carbon dioxide production (VCO_2_), respiratory exchange ratio (RER), and energy expenditure (kcal/day) were determined by indirect calorimetry, as previously described (Assaad et al. 2014).

### Tissue collection

After 14 days, NCD‐ and HFD‐fed animals were anesthetized with isoflurane. SOL and EDL muscles from the right leg were dissected and permeabilized for the in situ assessment of mitochondrial respiration and ROS production. The SOL and EDL muscles from the left leg were frozen in liquid nitrogen and stored at −80°C until they were used for the quantification of mRNA and protein contents.

### Mitochondrial function assessments

#### Preparation of permeabilized myofibers

Muscle samples were immediately immerged into ice‐cold stabilizing buffer A: 2.77 mmol/L CaK_2_ ethylene glycol‐bis‐(2‐aminoethylether)‐*N,N,N,N*‐tetraacetic acid (EGTA), 7.23 mmol/L K_2_ EGTA, 6.56 mmol/L MgCl_2_, 0.5 mmol/L dithiothreitol (DTT), 50 mmol/L 2‐(N‐morpholino) ethanesulfonic acid potassium salt (K‐MES), 20 mmol/L imidazol, 20 mmol/L taurine, 5.3 mmol/L Na_2_ ATP, and 15 mmol/L phosphocreatine, pH 7.3 at 4°C. Muscle samples were weighed and small fiber bundles were separated using fine forceps under a surgical dissecting microscope (Leica S4 E, Germany), as previously described by our group (Gouspillou et al. 2015, 2014; Picard et al. 2010). Fiber bundles were then incubated for 30 min at low rocking speed in buffer A supplemented with 0.05 mg/mL saponin to selectively permeabilize the sarcolemma. Fiber bundles were then washed three times for 10 min in buffer Z (110 mmol/L K‐MES, 35 mmol/LKCl, 1 mmol/L EGTA, 3 mmol/L MgCl2, and 10 mmol/L K2HPO4 pH 7.3 at 4°C, supplemented with 5 mg/mL BSA.

#### Mitochondrial respiration assay

The assessment of mitochondrial respiration in permeabilized myofiber was performed at 37°C using an oxygraph (Oxytherm; Hansatech Instruments). Briefly, 3 to 6 mg (wet weight) of permeabilized fiber bundles was added to 1 mL of buffer Z in the oxygraph chamber. Two different protocols were used for the assessment of mitochondrial respiration. The first protocol involved the sequential addition of the following substrates/inhibitors: 1–10 mmol/L glutamate + 5 mmol/L malate (G + M); 2–2 mmol/L ADP; 3–10 mmol/L Succinate (Succ); 4–10 *μ*mol/L antimycin A (AA). The second protocol involved the successive addition of the following substrates/inhibitors: 1–5 mmol/L malate + palmitoyl‐L‐carnitine 200 *μ*mol/L (M + PC); 2‐2 mmol/L ADP; 3–10 *μ*mol/L antimycin A (AA). Data derived from mitochondrial respiration experiments were analyzed using a homemade program developed using the Igor Pro software (Wavemetrics).

#### ROS production assay

Mitochondrial ROS production was evaluated in permeabilized myofibers by monitoring the rate of H_2_O_2_ release using the Amplex Red‐horseradish peroxidase (HRP) system. This was performed using a Hitachi FL‐2710 fluorescence spectrophotometer (Hitachi High Technologies Canada, Rexdale, ON, Canada) at excitation/emission wavelengths of 563/587 nm. Analyses were performed using the FL solutions software as previously described (Gouspillou et al. 2014; Picard et al. 2010). A standard curve was generated daily using known dilutions of H_2_O_2_. Permeabilized myofiber bundles (4–6 mg wet weight) were inserted in a thermojacketed, magnetically stirred cuvette containing 1000 *μ*L buffer Z, Amplex Red (5.5 *μ*mol/L), and HRP (1 U/mL) at 37°C. Mitochondrial H_2_O_2_ emission was assessed using two different protocols. The first protocol involved the sequential addition of the following substrates/inhibitors: 1‐glutamate + malate (10 + 5 mmol/L); 2‐ succinate (10 mmol/L); 3‐ADP (1 mmol/L); 5‐AA (10 *μ*mol/L). The second protocol involved the successive addition of the following substrates/inhibitors: 1‐malate (5 mmol/L) + palmitoyl‐L‐carnitine (200 *μ*mol/L); 2‐glutamate (10 mmol/L); 3‐ succinate (10 mmol/L); 4‐ADP (1 mmol/L); 5‐AA (10 *μ*mol/L). H_2_O_2_ emission assays were analyzed using a homemade program developed using the Igor Pro software (Wavemetrics).

### Immunoblots

Quantification of OPA1, Mfn2, and Drp1, as well as representative subunits of key proteins involved in mitochondrial oxidative phosphorylation (OXPHOS) were determined in SOL and EDL muscle homogenates in HFD and NCD rats as previously described (Leduc‐Gaudet et al. 2015). Approximately 30–40 mg of each muscle (SOL and EDL) was homogenized in 10 volumes of an extraction buffer containing 50 mmol/L Tris base, 150 mmol/L NaCl, 1% Triton X‐100, 0.5% sodium deoxycolate, 0.1% SDS, and 10 *μ*L/mL of a protease inhibitor cocktail (P8340; Sigma, St. Louis, MO). The homogenate was centrifuged at 15,000*g* for 15 min at 4°C. Protein content of the supernatant was determined using the Bradford method using BSA as standard. Aliquots of supernatant were mixed with Laemmli buffer and subsequently boiled at 95°C for 5 min. Approximately 30 *μ*g of proteins was loaded onto 8–12% gels, electrophoresed by SDS‐PAGE and then transferred to polyvinylidene fluoride membranes (Life Sciences). Membranes were incubated for 1 h at room temperature in a blocking buffer composed of 5% (w/v) nonfat dried milk in Tris‐buffered saline containing 0.1% Tween 20 (TBS‐T). Incubations were then carried out for 1 h with rabbit anti‐OPA1 (Abcam Ab42364; 1:1000), rabbit Mfn2 (Sigma, M6319; 1:1000), rabbit anti‐Drp1 (Cell Signaling, D6C7, 1:1000), and OXPHOS Blot (MitoSciences, MS6034; 1:500) antibodies diluted in blocking buffer. Membranes were washed six times for 5 min in TBS‐T and incubated with HRP‐conjugated secondary antibodies (Abcam, Ab6721 and Ab6728) diluted in blocking buffer for 1 h at room temperature. Signals were detected using enhanced chemiluminescence substrate (Biorad, Clarity ECL substrate, 170‐5060) and analyzed using ImageJ (NIH).

### RNA Extractions, cDNA Synthesis, and real‐time reverse transcription quantitative polymerase chain reaction

To determine the short‐term effect of HFD in the expression of several genes of interest, real‐time reverse transcription quantitative polymerase chain reaction (RT‐qPCR) analyses were performed. Skeletal muscle tissue samples were stored in RNAlater stabilization solution (Ambion) at −20°C. Between 15 and 60 mg of skeletal muscle tissue was homogenized in 1 mL of TRIzol Reagent (Ambion) with a TissueLyserII homogenizer (Qiagen Inc.,) and extracted according to Invitrogen's instructions. Samples were further processed using the PureLink RNA Mini Kit (Ambion) with contaminating DNA removed via DNase on column digestion. Total RNA samples used for RT‐PCR experiments were determined using the BioDrop spectrophotometer and purity estimated by 260∕280 nm absorption ratio. RNA integrity was evaluated by visualization of intact 18S and 28S RNA bands after agarose gel electrophoresis. cDNA was synthesized using Superscript Vilo Master Mix (Invitrogen) using 1 *μ*g of RNA per 20 *μ*L reaction. Real‐time PCR reactions were performed in triplicates in 384‐well plates in the QuantStudio6 (QuantStudio 6, Applied Biosystems Inc.,) using the Sybr Select Master Mix (Applied Biosystems, Inc.). Reaction conditions consisted of 2 *μ*L of a 1:10 cDNA dilution and 0.3 *μ*mol/L primers in a final volume of 10 *μ*L of supermix. The following cycling protocol was used: 2 min at 50 ᵒC, 2 min at 95ᵒC, followed by 40 cycles of 15 sec at 95°C, 15 sec at 58°C, and 1 min at 72°C. The specificity of the reaction was verified by a melting curve analysis. Negative RT controls and no template controls were included in the PCR runs. Reference genes were identified by running TaqMan array Rat Endogenous Control plates (Applied Biosystems Inc.) with three normal and three high‐fat samples. Of the 32 genes, four with the best gene expression stability measure (Expression Suite, Applied Biosystems Inc.) were selected for normalization: Pum1, Pgk1, Psmc4, and Rpl30 for the EDL, and Pgk1, Rpl30, Ppib, and Pum1 for the SOL. Primers used (Table S2) were designed using Primer Blast (NCBI). Whenever possible intron spanning primers were selected to avoid amplification of genomic DNA. A standard curve obtained by scalar dilution of a cDNA pool was always generated to verify PCR efficiency. Normalization and relative amounts of messenger RNA (mRNA) were calculated using Data Assist v3.01 (Applied Biosystems Inc.) and expressed as the fold change in the HFD group compared to the control (normal diet) using the ΔΔCt value procedure.

### Data analysis and statistics

Unless otherwise mentioned in figure legends, all data are reported as means ± SEM of 8–10 animals per group and comparison between NCD and HFD rats were performed using unpaired two‐tailed Student's test. Results from RT‐qPCR were quantified by the Data Assist v3.01 (Applied Biosystems Inc.) sequence detection software and normalized by four housekeeping genes. The relative changes in gene expression were calculated using ΔΔCt method as described in (Livak and Schmittgen 2001). Results were considered significantly different when *P < *0.05. All statistical analyses were performed using GraphPad Prism (GraphPad Software 6).

## Results

### Animal characteristics

**Figure 1:**
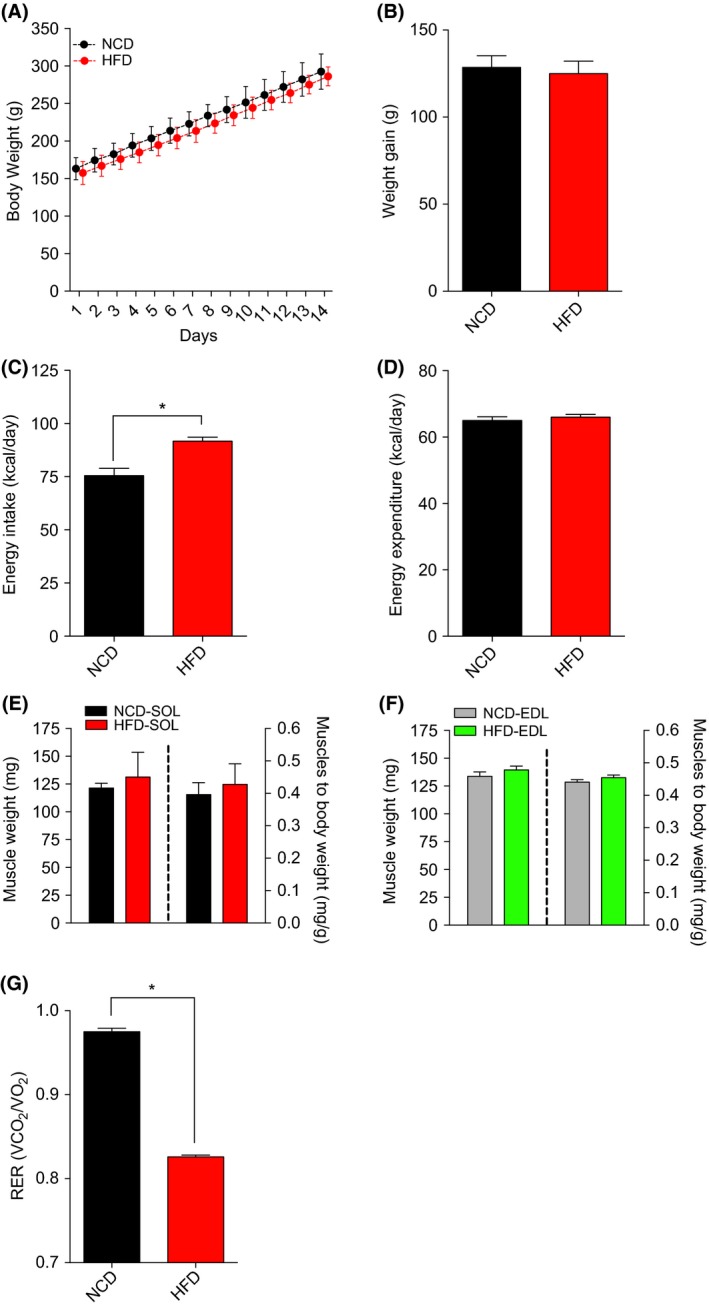
The impact of NCD and HFD on animal characteristics. Changes in body weight (A), total weight gain (B), Energy intake (ND: Daily Food Intake (g) X 2.89 kcal/day; HFD: Daily Food Intake (g) X 4.80 kcal/day) (C), Energy Expenditure (kcal/day) (D), weights of the oxidative SOL (C) and glycolytic extensor digitorum longus. (D) muscles in young rats submitted to NCD or HFD diets for 14 days: (E) Average values of respiratory exchange ratio recorded over 14 days. Data are presented as means ± SEM;*P < 0.05 by comparing NCD to HFD rats (*n *= 8–18 per group).NCD, normal chow; HFD, high fat diet.

No difference in body at weight was observed between HFD and NCD rats at baseline. Fourteen days of HFD did not alter the normal weight gain seen in NCD animals. (Fig. 1A–B), although HFD rats ingested higher amounts of energy (kcal) than their NCD counterparts (Fig. 1C). There was no significant difference in energy expenditure between both groups (Fig. 1D).No difference in the absolute and relative weights of the SOL and EDL muscles was observed between HFD and NCD rats (Fig. 1E and F). As expected, the RER was significantly lower in HFD versus NCD rats (Fig. 1G) indicating a higher reliance on lipid substrates.

### Short‐term effects of high fat and normal chow diets on mitochondrial respiration rate

**Figure 2:**
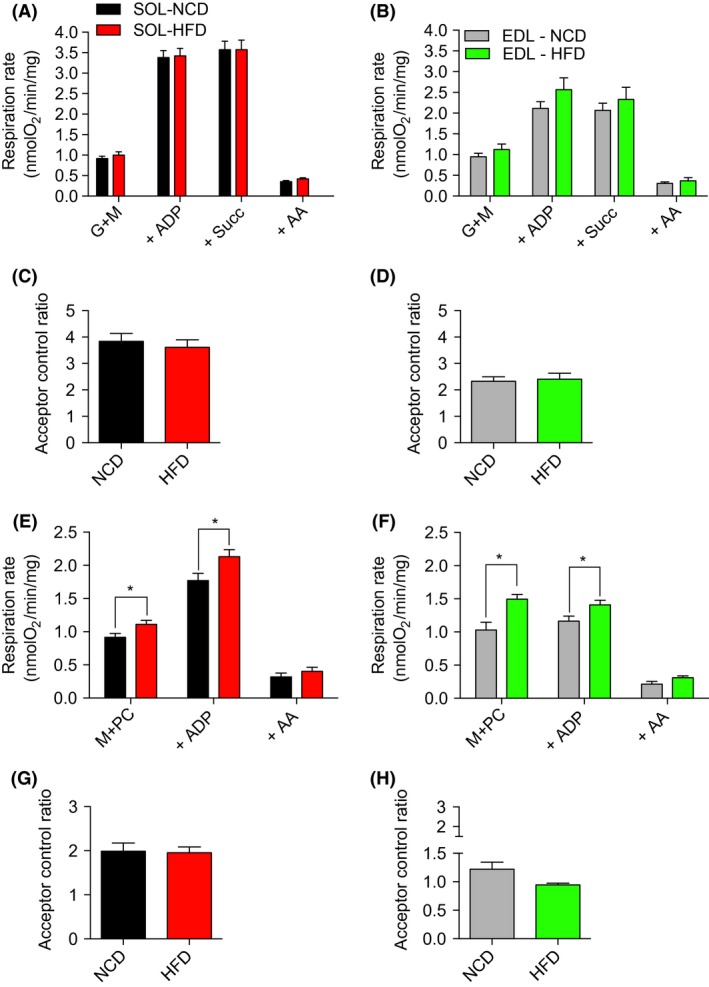
Parameters related to mitochondrial respiration in permeabilized myofibers from the oxidative SOL and the glycolytic EDL skeletal muscles from young rats submitted to 14 days of NCD or HFD. Results were normalized per mg of muscle. (A, B) Mitochondrial substrates and inhibitors were subsequently added as followed: glutamate + malate (G + M), ADP, antimycin A (AA). (C, D) ACR, a parameter indicative of mitochondrial coupling efficiency, was determined by dividing the respiration rate with GM + ADP (ADP, state 3) by the respiration rate with GM (state 2). (E, F) Mitochondrial substrates and inhibitors were subsequently added as follows: malate + palmitoly‐L‐carnitine (M + PC), ADP, glutamate (G), succinate (SUCC), antimycin A (AA). (G, H) The acceptor control ratio (ACR) was determined by dividing the respiration rate with GM + PC + ADP (ADP, state 3) by the respiration rate with M + PC (state 2). Data are presented as means ± SEM; *P < 0.05 by comparing HFD to NCD rats (*n *= 8–10 per group). EDL, extensor digitorum longus; NCD, normal chow; HFD, high‐fat diet; EDL, extensor digitorum longus.

When mitochondria were treated with complex I substrates (glutamate + malate), no difference in basal (state 3; ADP restricted respiration) and maximal (state 3; ADP‐stimulated respiration) mitochondrial respiration was observed between HFD and NCD groups in both SOL and EDL muscles (Fig. 2A and B). Similarly, no difference in the state 3 respiration rate driven by complex I and II substrates (G + M + Succ + ADP) was observed between HFD and NCD groups in both SOL and EDL muscles (Fig. 2A and B). No difference in the acceptor control ratio (ACR; Fig 2C and D), an index of the phosphorylation coupling efficiency. When mitochondria were provided with lipid substrates (malate + palmitoyl‐L‐carnitine) both basal and maximal respiration rates were significantly increased in SOL and EDL muscles of HFD versus NCD rats (Fig. 2E and F). No difference in the ACR was observed when malate + palmitoyl‐L‐carnitine were used as substrates (Fig. 2G and H).

### Impact of short‐term high‐fat on mitochondrial H_2_O_2_ emission

**Figure 3:**
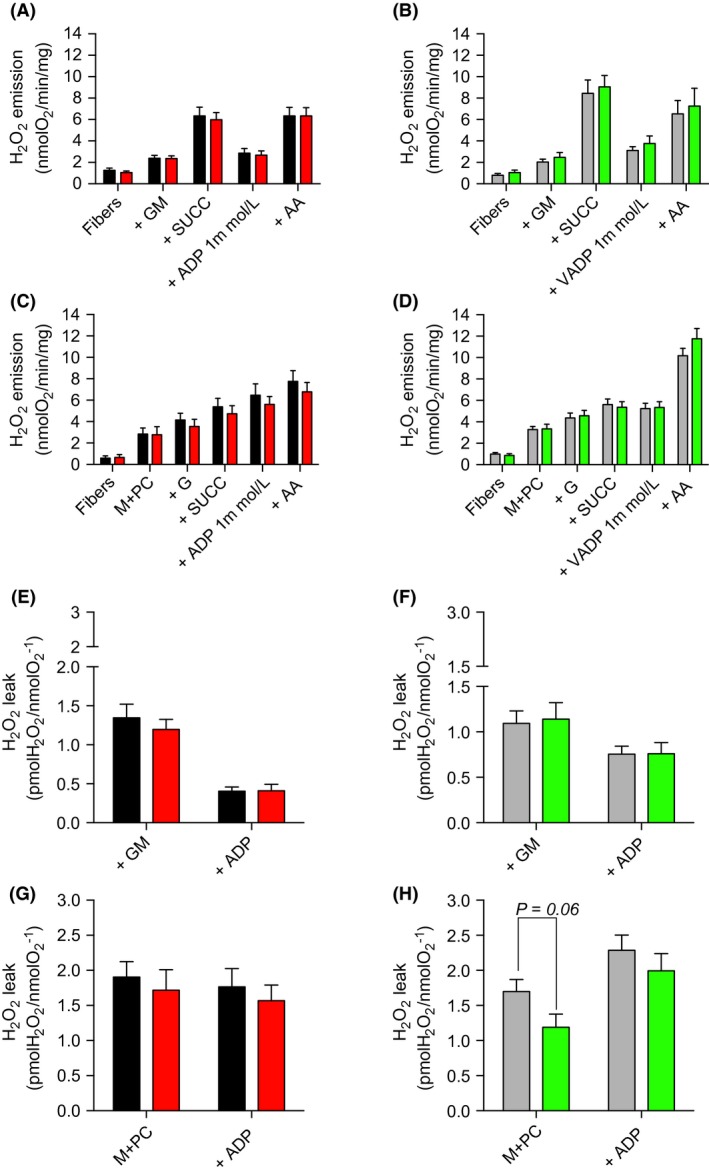
Mitochondrial H_2_O_2_ production in permeabilized myofibers prepared from the oxidative SOL and the glycolytic EDL muscles in young rats submitted to NCD or HFD. (A, B) Mitochondrial substrates and inhibitors were subsequently added as follows: glutamate + malate (G + M), succinate (+SUCC), ADP, antimycin A (AA). (C, D) Mitochondrial substrates and inhibitors were subsequently added as follows: Malate + palmitoyl‐L‐carnitine (M + PC), glutamate (G), succinate (SUCC), ADP, antimycin A (AA). (E–H) Mitochondrial‐free radical leak was determined by dividing H_2_O_2_ emission rates by their corresponding respiration rates (using data presented in Figure 2A, B, C, and D). Data are presented as means ± SEM;*P < 0.05 by comparing HFD versus NCD rats (*n *= 8–12 per group). EDL, extensor digitorum longus.

Maximal H_2_O_2_ emission was assessed in permeabilized myofiber using the amplex red – HRP system. As described in Figure 3A–D, no difference in H_2_O_2_ emission was observed between HFD and NCD rats in the EDL and SOL under all tested conditions. Furthermore, mitochondrial ROS leak, calculated by dividing H_2_O_2_ emission rates by their corresponding respiration rates, was also not altered by HFD (Fig. 3E–H).

### Effects of a short‐term high‐fat diet on mitochondrial protein content

**Figure 4:**
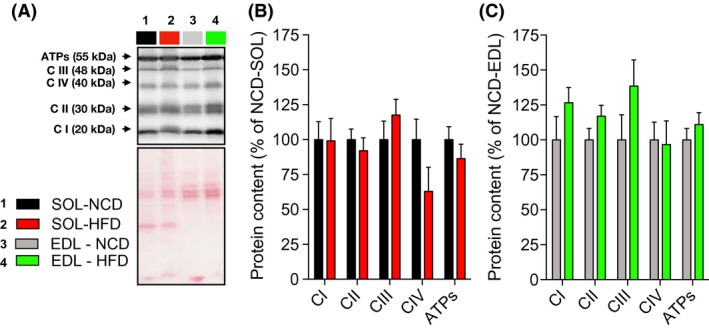
Oxidative phosphorylation protein contents in oxidative (SOL) and glycolytic (EDL) skeletal muscles of young rats submitted to 14 days of NCD or HFD. (A) Immumoblot for representative subunits of complexes I, II, III, IV, and ATP synthase obtained in NCD and HFD rats and corresponding ponceau stains for normalizing quantifications. Quantifications of representative subunits of complexes I, II, III, IV, and ATP synthase protein contents in SOL (B) and EDL (C) muscles of NCD and HFD rats (*n *= 6–8 for each group). Results are presented as mean ± SEM. EDL, extensor digitorum longus

Protein quantification of representative subunits of complexes I, II, III, IV, and ATP synthase was determined using immunoblots (Fig. 4 **A**). As presented in Figure 4B (SOL) and 4C (EDL), the abundance of oxidative phosphorylation (OXPHOS) proteins was not altered in HFD rats.

### Effects of a short‐term high‐fat diet on the mRNA levels of uncoupling proteins, Mn‐SOD, proteins involved in mitochondrial lipid importation and in mitochondrial dynamics

**Figure 5:**
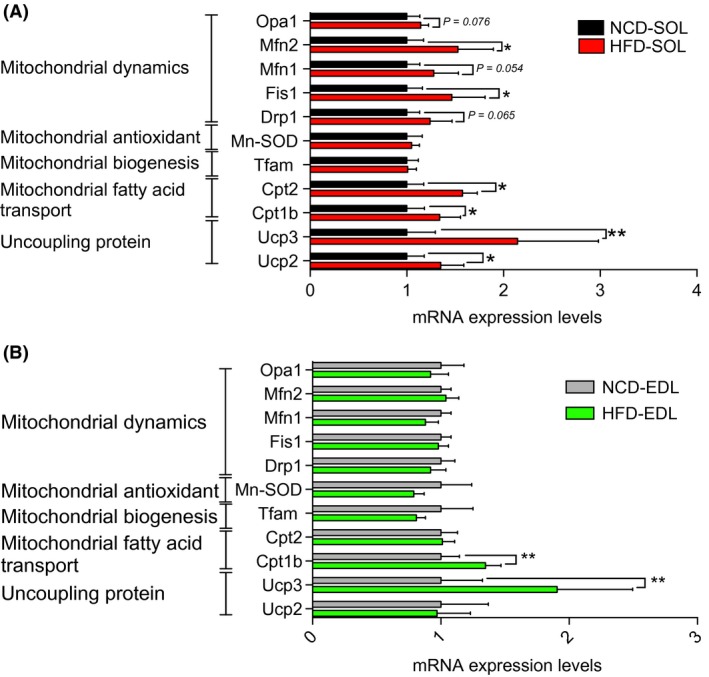
Expression levels of genes involved in mitochondrial fatty acid transport, antioxidant defense and dynamics in the oxidative SOL (A) and the glycolytic extensor digitorum longus (B) skeletal muscles in young rats submitted to 14 days of NCD or HFD. Results are presented as fold changes with 95% intervals of confidence in HFD versus NCD rats. *P < 0.05 (*n *= 10–14 per group).NCD, normal chow; HFD, high fat diet.

As shown in Figure 5A, Ucp2 (~35%; *P = *0.019), UCP3 (~114%; *P = *0.002), CPT1b (~34%; *P < *0.019), and CPT2 (~57%; *P < *0.001) mRNA levels were upregulated in oxidative (SOL) skeletal muscles of HFD rats. In the EDL, the expression of UCP3 (~90%; *P = *0.007) and CPT1b (~34%; *P = *0.004) mRNA was significantly higher in HFD rats (Fig. 5B). Interestingly, no difference in Mn‐SOD expression was observed between NCD and HFD rats in both the EDL and SOL, suggesting that mitochondrial antioxidant mechanisms are not upregulated by HFD in young rats. As shown in Figure 5A, higher mRNA expression of Fis1 (~46%; *P = *0.013) and Mfn2 (~52%; *P = *0.013) was observed in SOL of HFD rats. However, no difference in the expression level of protein involved in mitochondrial dynamics was detected for EDL (Fig. 5B).

### Effects of high‐fat diet on mitochondrial dynamics protein content

**Figure 6:**
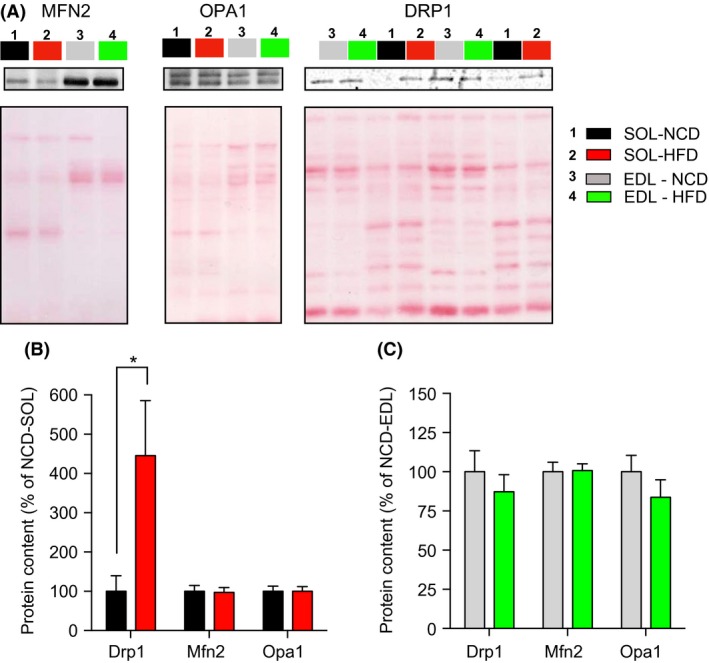
Impact of a short‐term HFD on the content of protein involved in mitochondrial dynamics in the oxidative SOL and glycolytic EDL muscles after 14 days of HFD or NCD. (A) Representative immunoblots of OPA1, Mfn2, and Drp1 as well as their corresponding ponceau stains. Quantification of OPA1, Mfn2, and Drp1 protein contents (normalized for their corresponding ponceau stain intensities) in SOL (B) and EDL (C) muscles of NCD and HFD rats (*n *= 6–8 in each group). Data are presented as Mean ± SEM. *P < 0.05. EDL, extensor digitorum longus; NCD, normal chow; HFD, high‐fat diet.

As can be seen in Figure 6A, no difference in Mfn2 and OPA1 content were observed between NCD and HFD rats in both the SOL (Figure 6B) and EDL (Fig. 6C) muscles. Although DRP1 content was not altered in the EDL (Fig. 5C), DRP1 protein content was increased by over fourfold in the SOL (Fig. 6B) of HFD rats.

## Discussion

The aim of this study was to investigate the short‐term effects of HFD on a host of mitochondrial functions in oxidative and glycolytic skeletal muscle types of young rats. Our main results show that a short‐term HFD enhances mitochondrial capacity to oxidize fatty acids without altering mitochondrial content, H_2_O_2_ emission and coupling efficiency in both oxidative (SOL) and glycolytic (EDL) skeletal muscles. They also highlight that the impact of HFD on proteins regulating mitochondrial dynamics differ between oxidative and glycolytic skeletal muscles. Importantly, these HFD‐induced changes in mitochondrial function occurred in the absence of changes in body weight, suggesting that the HFD‐induced adaptation of mitochondrial metabolism precedes the development of clinical obesity.

### The impact of HFD on mitochondrial respiration and content in young animals

The increased mitochondrial capacity to oxidize fatty acids in response to HFD observed in this study (Fig. 2) supports and extends findings from the only previous study proposing, under the same treatment, that palmitate oxidation is enhanced in the tibialis anterior of young animals (Hancock et al. 2008). In their study, Hancock et al. have suggested that, in response to HFD, the upregulation of palmitate oxidation involves an increase in mitochondrial content. This observation is not in line with our present findings. Indeed, HFD had no influence on mitochondrial respiration when classical complex I and II substrates were used to feed the electron transport chain. Furthermore, HFD did not alter the content of representative subunits of the complexes of oxidative phosphorylation. If mitochondrial content was increased, one could have hypothesized that complex I and complex II driven respiration and the content of proteins involved in the oxidative phosphorylation would have been increased (Figs. 2 and 4). Following the same rationale, it is very unlikely that any HFD‐induced posttranslational modification of the complexes involved in the oxidative phosphorylation could explain the increased mitochondrial capacity to oxidize fatty acids in response to HFD since the state 3 respiration driven by complex I and II substrates were unaltered by high‐fat feeding. However, our present results suggest that in response to HFD, the enhanced mitochondrial capacity to oxidize fatty acids is caused by the increased expression of genes involved in mitochondrial lipid transport, such as CPT1 (Fig. 5). It is also possible that HFD could trigger the expression of beta‐oxidation enzymes. This view is supported by the increase in beta‐hydroxyacyl CoA dehydrogenase activity previously reported in response to HFD (Li et al. 2016). It is important to note that the absence of effect of HFD on mitochondrial content is not necessarily contradicting the results from Hancock et al. Indeed, Hancock et al. submitted their animals to HFD for a period that was two times longer than in our experimental protocol. This suggests that in response to HFD, mitochondrion of skeletal muscles could undergo prompt transitory adaptations (i.e., within the first 2 weeks) to increase their intrinsic capacity to import and oxidize fatty acids. If high‐fat feeding is maintained up to 4 weeks, it could then trigger mitochondrial biogenesis.

Higher mRNA expression of genes regulating proton leak (UCP2, UCP3) was observed in response to HFD in glycolytic and oxidative skeletal muscles (Fig. 5). Our results are therefore in line with the increase in gene and protein expression of UCPs that was reported in SOL and gastrocnemius muscles in adult mice submitted to HFD for 18 weeks (Felipe et al. 2003). It is, however, important to note that HFD did not alter the acceptor control ratio (ACR, a marker of mitochondrial coupling efficiency; Figure 2), therefore indicating that this increase in UCP2 and 3 expression is not associated to the alteration of proton conductance of the mitochondrial inner membrane. These results are in line with those of previous studies showing no difference in total oxygen consumption following UCP3 inactivation in mice (Vidal‐Puig et al. 2000; Gong et al. 2000) and those suggesting that UCP2 and 3 are not involved in basal proton conductance (Echtay et al. 2002; Brand and Esteves 2005). It was, however, reported that the uncoupling activity of UCP2 and 3 can be activated, notably in the presence of alkenals generated during oxidative stress such as 4‐hydroxynonenal (Echtay et al. 2002). Under most conditions, uncoupling is associated with a reduction in mitochondrial ROS generation (Brand 2000). Therefore, the observed increase in UCP 2 and 3 may represent a protective mechanism conferring mitochondria a greater capacity to attenuate their ROS production if the later becomes excessive.

### The impact of HFD on mitochondrial ROS production in young animals

ROS overproduction is often proposed to promote insulin resistance in response to HFD in adult skeletal muscles (Anderson et al. 2009; Di Meo et al. 2017). Notably, Anderson et al. (2009) reported that HFD increases mitochondrial H_2_O_2_ emission in adult mice. Furthermore, they have also shown that in response to HFD, insulin signaling was completely normalized by a mitochondrial‐targeted antioxidant in rats and by the overexpression of a mitochondrial‐targeted catalase in mice (Anderson et al. 2009). In contrast with these findings, no impact of HFD on H_2_O_2_ emission in skeletal muscle of young rats was observed in this study (Fig. 3). In line with this result, HFD did not alter SOD2 expression (Fig. 5), an antioxidant enzyme known to be upregulated in response to ROS overproduction and oxidative stress (Candas and Li 2014). These discrepancies between our results and those from Anderson et al. could be explained by the differential impact of a short‐term HFD on mitochondrial ROS production in developing versus adult skeletal muscles. Further studies are required to test this hypothesis.

### The impact of HFD on proteins regulating mitochondrial dynamics in young animals

Mitochondria have the capacity to alter their morphology through processes termed fusion and fission (Chan 2006). This is collectively defined as mitochondrial dynamics. Mitochondrial dynamics play an important role in the maintenance of mitochondrial functions in the skeletal muscle and, as highlighted in the introduction, recent studies have suggested in adult animals that impairments in mitochondrial dynamics could be implicated in HFD‐induced insulin resistance (Bach et al. 2005; Chan 2012; Cheng and Almeida 2014; Sebastian et al. 2012; Putti et al. 2015; Jheng et al. 2012). Here, we show that exposing young rats to a short‐term HFD does not alter mRNA/protein contents of OPA1, Mfn2, and Drp1 in the glycolytic EDL muscle (Figs. 5 and 6). In contrast, we observed in the SOL that HFD (1) upregulated the expression of Mfn2 and Fis1 and (2) triggered a fourfold increase in Drp‐1 content without affecting Mfn2 and OPA1 content (Figs. 5 and 6). This increase in Drp1 content in the SOL suggests that a short‐term HFD could trigger mitochondrial fragmentation in young oxidative skeletal muscles. This conclusion is in line with those of a previous study suggesting that the treatment of differentiated C2C12 myocytes with palmitate increases Drp1 expression and triggers mitochondrial fragmentation (Jheng et al. 2012). Increased mitochondrial fission was also reported in the adult skeletal muscles and liver of mice with obese (*ob/ob, db/db*) genotypes (Jheng et al. 2012; Holmstrom et al. 2012). In response to a highly saturated HFD, mitochondrial fission was stimulated through the upregulation of Drp1 and Fis1 expression in rat liver and mouse gastrocnemius muscle (Lionetti et al. 2014; Liu et al. 2014). Our results highlight that in response to HFD, the increase in Drp1 content is an early event in oxidative skeletal muscle of young rats. More studies are required to fully elucidate the consequences of this early increase in Drp1 content. However, it is appealing to hypothesize that this early increase in Drp1 expression could result in mitochondrial fragmentation which could, when HFD is prolonged, contribute to mitochondrial dysfunction and the development of insulin resistance.

## Conclusions

Taken together, the results of this study provide evidence that a short‐term HFD enhances mitochondrial capacity to oxidize fatty acids in both oxidative and glycolytic skeletal muscles without altering ROS production in young rats. This improvement in mitochondrial function is not associated with an increase in markers of mitochondrial content, but with an upregulation of several key genes involved in lipid metabolism and uncoupling. They also indicate that a short‐term HFD in developing oxidative skeletal muscle increases Drp1 content.

## Conflict of Interest

None declared.

## Supporting information




**Table S1:** Detailed composition of the NCD and HFD.
**Table S2:** Primer Sequence Used for RT‐qPCR Amplification.
