# Supplementary material for: The impact of a short‐term high‐fat diet on mitochondrial respiration, reactive oxygen species production, and dynamics in oxidative and glycolytic skeletal muscles of young rats
Source: Physiol Rep. 2018 Feb 26;6(4):e13548. doi: 10.14814/phy2.13548 (PMC6430054; doi:10.14814/phy2.13548)
Supplement: Supplementary file 1 — Table S1: Detailed composition of the NCD and HFD. Table S2: Primer Sequence Used for RT‐qPCR Amplification. [file PHY2-6-e13548-s001.docx]

**Supplemental Table 1: Detailed composition of the NCD and HFD**

|  | | **NCD^A^** | | **HFD**^B^ | |  |
| --- | --- | --- | --- | --- | --- | --- |
| **Physiological fuel value**^C^ | **kcal/g** | | | | **kcal/g** | |
|  | 2.89 | | | | 4.80 | |
| **Macronutrients**^C^ | **% kcal** | | | | **% kcal** | |
| Protein | 21.4 | | | | 19 | |
| Fat | 13 | | | | 61.8 | |
| Carbohydrates | 65.6 | | | | 19.2 | |
| **Approximate fat content** | | | **% of total fat^D^** | | **% of total fat^E^** | |
| Saturated fat | | | 30 | | 32 | |
| Monounsaturated fat | | | 38.5 | | 36 | |
| Polyunsaturated fat | | | 31.5 | | 32 | |
| **Ingredients** | | | **Representative ingredients**  **(or g% when available)** | | **g%** | |
| Lard | | | - | | 31.7 | |
| Casein | | | - | | 25.8 | |
| Maltodextrin | | | - | | 16.2 | |
| Sucrose | | | - | | 9.9 | |
| Fibers (digestible + non-digestible) | | | 20.7 | | 6.4 | |
| Soybean oil | | | - | | 3.2 | |
| Potassium citrate | | | - | | 2.1 | |
| Dicalcium phosphate, 1 H_2_O | | | Yes | | 1.7 | |
| Mineral mix | | | Yes | | 1.3 | |
| Vitamin mix | | | Yes | | 1.3 | |
| Calcium carbonate | | | Yes | | 0.7 | |
| L-Cystine | | | 0.3 | | 0.4 | |
| Choline bitartrate | | |  | | 0.3 | |
| Corn | | | Yes | | - | |
| Wheat middlings | | | Yes | | - | |
| Wheat | | | Yes | | - | |
| Dehulled soybean meal | | | Yes | | - | |
| Fish meal | | | Yes | | - | |
| Poultry fat | | | Yes | | - | |
| Whey | | | Yes | | - | |
| Salt | | | Yes | | - | |
| Amino acids | | | Yes | | - | |

^A^ Nutrient composition of the standard chow diet (Charles River Rodent Diet # 5075, Cargill Animal Nutrition, Minnetonka, MN). ^B^ Commercial HFD diet (D12492 diet, Research Diets Inc., USA) prepared using purified food-grade elements. ^C^ Physiological fuel value (kcal/g) calculated using modified Atwater factors (3.5kcal to protein, 8.5kcal/g to fat and 3.5kcal/g to carbohydrate.) for both diets. ^D^ Percent saturated and unsaturated (monounsaturated and polyunsaturated) fat values as previously described by Ysari *et al*., Mol Cell Biochem 335:291-9. ^E^ Percent saturated and unsaturated fat values as previously described by Guyenet SJ *et al.,* Brain research 1512:97-105.

9**.**

**Supplemental Table 2: Primer Sequence Used for RT-qPCR Amplification**

| *Target gene* | *Organism* | *Forward primer 5’-----------˃ 3’*  *Reverse primer 5’-----------˃ 3’* | *Accession number* |
| --- | --- | --- | --- |
| *UCP3* | Rat | AAAGACCCGATACATGAACGCT  AAGGAGGGCATGAATCCTTTGT | NM_013167.2 |
| *CPT1B* | Rat | TGGGCAACCAACTATGTAAGTGA  TGCTTGTTGGCTCGTGTTCTTA | NM_013200.1 |
| CPT2 | Rat | GATTATCTGCAGCACAGCATCG  CTTCTGTTCTCCTGAACTGGCT | NM_012930.1 |
| Dnm1l | Rat | TAATAAGGGAGTAAGCCCTGAGC  AAGCTCAATATCCTTGGGCTGA | NM_053655.3 |
| Fis1 | Rat | ACAATGACGACATCCGTAGAGG  GCCTTTTCATATTCCTTGAGCCG | NM_001105919.1 |
| Mfn1 | Rat | CTCGGAATCAACGCTGATGAAC  TGCGCACATCCTCCATATATTCT | NM_138976.1 |
| Mfn2 | Rat | TATAAGAATGAACTGCACCGCCA  CACAGGAAGAAGGGGCTTCAA | NM_130894.4 |
| Opa1 | Rat | GAGGATCCGGTGAGATGATGAC  CTGCAAGATCTTCCTCCTTGGT | NM_133585.3 |
| Tfam | Rat | GCTAAACACCCAGATGCAAAAGT  GCTCACAGCTTCTTTGTACACC | NM_031326.1 |
| Mn SOD | Rat | TGGCTTCAATAAGGAGCAAGGT  AATCCCCAGCAGTGGAATAAGG | NM_017051.2 |
